# Author Correction: A randomized controlled trial of pharmacist-led therapeutic carbohydrate and energy restriction in type 2 diabetes

**DOI:** 10.1038/s41467-022-30330-7

**Published:** 2022-05-05

**Authors:** Cody Durrer, Sean McKelvey, Joel Singer, Alan M. Batterham, James D. Johnson, Kelsey Gudmundson, Jay Wortman, Jonathan P. Little

**Affiliations:** 1 School of Health and Exercise Sciences, University of British Columbia, Kelowna, BC Canada; 2 Institute for Personalized Therapeutic Nutrition, Vancouver, BC Canada; 3 School of Population and Public Health, University of British Columbia, Vancouver, BC Canada; 4 Centre for Rehabilitation, School of Health and Life Sciences, Teesside University, Middlesbrough, United Kingdom; 5 Diabetes Research Group, Life Sciences Institute, Faculty of Medicine, University of British Columbia, Vancouver, BC Canada; 6 Faculty of Medicine, University of British Columbia, Vancouver, BC Canada

**Keywords:** Type 2 diabetes, Nutrition therapy

Correction to: *Nature Communications* 10.1038/s41467-021-25667-4, published online 10 September 2021.

The original version of this article contained errors in Tables 1 and 2, where units of gamma-glutamyl transferase (GGT), aspartate aminotransferase (AST), and alanine aminotransferase (ALT) were expressed as mmol/L, while they should have been expressed as U/L. This has been corrected in both the PDF and HTML versions of the Article.

