# Supplementary material for: A randomized controlled trial of pharmacist-led therapeutic carbohydrate and energy restriction in type 2 diabetes
Source: Nat Commun. 2021 Sep 10;12:5367. doi: 10.1038/s41467-021-25667-4 (PMC8433183; doi:10.1038/s41467-021-25667-4)
Supplement: Supplementary file 1 — Supplementary Information File [file 41467_2021_25667_MOESM1_ESM.pdf]

Supplementary Information File

A randomized controlled trial of pharmacist-led therapeutic carbohydrate and energy restriction in type 2 diabetes

*To whom correspondence should be addressed:

Dr. Jonathan Little

School of Health and Exercise Sciences

University of British Columbia

Kelowna, BC V1V 1V7

CANADA

Ph: +1-250-807-9876

Email: jonathan.little@ubc.ca

Cody Durrer[1], Sean McKelvey[2], Joel Singer[3], Alan M. Batterham[4], James D. Johnson[2,5], Kelsey Gudmundson[1], Jay Wortman[6], Jonathan P. Little[*1,2]

1 School of Health and Exercise Sciences, University of British Columbia, Kelowna, BC CANADA

2 Institute for Personalized Therapeutic Nutrition, Vancouver, BC CANADA

3 School of Population and Public Health, University of British Columbia, Vancouver, BC CANADA

4 Centre for Rehabilitation, School of Health and Life Sciences, Teesside University, Middlesbrough, UNITED KINGDOM

5 Diabetes Research Group, Life Sciences Institute, Faculty of Medicine, University of British Columbia, Vancouver, BC CANADA

6 Faculty of Medicine, University of British Columbia, BC CANADA

Supplementary Table 1: Subgroup analyses of the primary outcome

	Proportion (%)	p value
Sex		
Male	42.5 (25.3 to 57.1)	
Female	33.3 (19.5 to 45.8)	
Difference	9.2 (-10.8 to 28.4)	0.37
Insulin use at baseline		
Yes	33.3 (14.7 to 49.7)	
No	36.6 (25.4 to 46.9)	
Difference	-3.3 (-23.9 to 17.6)	0.76

Data are the proportion achieving the primary outcome within each subgroup and the corresponding difference in proportions with their respective 95% confidence intervals. Data were analyzed using a generalized linear model with a binomial distribution and logit link, with the Huber/White/sandwich variance estimator.

Supplementary Table 2: Treatment effect on HbA1c and Weight disaggregated by sex

	Treatment Effect	p value
HbA1c		
Sex		
Male	-1.6 (-2.1 to -1.1)	
Female	-1.2 (-1.7 to -0.7)	
Difference	-0.4 (-1.0 to 0.2)	0.21
Weight		
Sex		
Male	-14.0 (-16.0 to -12.0)	
Female	-10.5 (-12.3 to -8.6)	
Difference	-3.5 (-5.6 to -1.5)	0.0012

Data are treatment effect estimates in each subgroup (i.e., Male or Female) and the corresponding difference in treatment effect with their respective 95% confidence intervals. Data were analyzed using constrained baseline longitudinal analysis via linear mixed models

Supplementary Table 3: Glucose-lowering medication changes

Medication Class	Deprescribed	Increased	Lowered	No Change	n
Pharm-TCR					
Insulin	56	0	44	0	27
Sulfonylurea	84	0	10	6	31
SGLT2 Inhibitor	92	0	8	0	13
TZD	100	0	0	0	1
DPP4 Inhibitor	87	0	7	7	15
GLP1 Agonist	62	12	0	25	15
Biguanide	36	1	25	38	78
TAU					
Insulin	0	8	8	85	13
Sulfonylurea	5	15	0	80	20
SGLT2 Inhibitor	0	10	0	90	10
TZD	0	0	0	100	1
DPP4 Inhibitor	0	0	0	100	8
GLP1 Agonist	0	9	0	91	11
Biguanide	2	6	2	91	64

Medication changes are denoted as percentages of participants taking each respective medication at baseline. SGLT2: Sodium-glucose transport protein 2; TZD: Thiazolidinedione; DPP4: Dipeptidyl peptidase-4; GLP1: Glucagon-like peptide-1.

Supplementary Table 4: Blood pressure medication changes

Medication Class	Deprescribed	Increased	Lowered	No Change	n
Pharm-TCR					
ACE Inhibitors	11	8	25	56	36
ARBs	37	0	11	53	19
Beta Blockers	25	0	25	50	8
Calcium Channel Blockers	11	6	17	67	18
Diuretics	58	0	12	29	24
Vasodilators	0	0	0	100	1
TAU					
ACE Inhibitors	0	0	0	100	23
ARBs	0	5	5	90	21
Beta Blockers	0	10	0	90	10
Calcium Channel Blockers	0	0	0	100	14

Diuretics	0	0	0	100	20

Medication changes are denoted as percentages of participants taking each respective medication at baseline. ACE: Angiotensin-converting enzyme; ARB: Angiotensin II receptor blockers.

Supplementary Table 5: Macronutrient and kilocalorie intake at baseline, week 6, and week 12

	Kilocalories	Kilojoules	Carbohydrates (g)	Fats (g)	Protein (g)
Pharm-TCR					
Baseline	1789 (543)	7485 (2272)	188 (72)	72 (29)	85 (30)
Week 6	989 (217)	4138 (906)	69 (22)	34 (14)	106 (22)
Week 12	984 (212)	4116 (885)	66 (20)	34 (15)	106 (23)
TAU					
Baseline	1806 (608)	7558 (2544)	192 (80)	76 (32)	87 (28)
Week 6	1714 (597)	7172 (2497)	174 (71)	71 (37)	90 (28)
Week 12	1667 (589)	6975 (2465)	166 (85)	74 (45)	90 (28)

Data are daily means (SD). Descriptive data are based on complete cases of n=67 (Pharm-TCR) and n=53 (TAU).
